# Camp Diarrhœa, near Vicksburg

**Published:** 1863-08

**Authors:** Geo. Winch

**Affiliations:** Assistant Surgeon 29th Reg. Wis. Vol.


### CAMP DIARRHEA, NEAR VICKSBURG.

*June 22nd*, 1863.

Being in camp here about five weeks, crowded into a ravine, the men prevented from having the atmosphere of the hill tops by the enemies sharp shooters, and not procuring sufficient and suitable rations until quite lately, and being without sufficient tents and clothing to prevent them from the cold and dampness of the night, when Gen. Grant's army were on the march from Grand Gulf to this place, with all their fighting and fatigue—is it strange, that we should have many cases of Camp Diarrhea? Most cases in this regiments complain of pain across the abdomen below the umbilicus.

In some cases it is very severe, accompanied with tympanitis; others quite light. It is seldom a continual pain. There are some dysenteric cases where pain is lower down. Pulse in most cases natural, but not always. When there is pain in the epigastric region and at the same time vomiting, it is obstinate, unless promptly treated. There are but very few cases of this kind. I have noticed three different grades of the tongue. Sometimes covered with a yellow coat, at other times fissured with short deep fissures. The tongue in this case is large pale and flabby. Again having almost a natural appearance. Having charge of the Regiment in the field, I am obliged to depend on patients concerning the color of their stools which varies more than any one symptom. Some are green, others dark, some tell me their stools look like water.

There have been a number of cases within the last few days with hemorrhage from the bowels, without much pain. The stools are large and frequent, it soon brings on lassitude and debility, unless checked in which I have found, no difficulty. But I am informed in another brigade, near by, many such cases prove fatal.

It is not dysentery, it wants the pain, fever, and other symptoms. In all cases the powers of digestion are weak. Food of easy digestion passes the bowels as taken into the stomach. The treatment must vary. In some cases with a clean tongue, with or without pain over the abdomen, opium from one to two grain doses, combined with either alum, tannin, or plumbi acetatis, has a good effect without any other treatment. In cases where alum was used, there was less pain and bloating after the diarrhea was checked. In those cases where there is severe pain with tympanitis, and the tongue covered with a yellow coat, the following powder has satisfied me, Calomel grs. x. to xv. bi-carb. soda grs. x. to xv. Ipecac gr. iss. M. The bowels move free with less pain. They complain no more of headache, &c., and, many cases without further treatment are well in a short time. But after this, if necessary, opium and astringents cure, if given before, increase the difficulty. In those rare cases of obstinate vomiting the ancient powder of small doses of Calomel once in two hours, operate well; combined if the stools are green with bi-carb soda. When accompanied by hæmorhage the following powder has (in my cases) controlled it: Opium gr. j. Pulv. Camphor grs. ij. Acetat of Lead grs. iij. M. Leaving the patient with some pain but it would not last long.

Army surgeons are well aware, they have not under their control (for the privates), the most essential remedy, *good diet.*

GEO. WINCH, M. D.,

Assistant Surgeon 29th Reg. Wis. Vol.

